# Supplementary material for: Gold Nanoclusters-Based NIR-II Photosensitizers with Catalase-like Activity for Boosted Photodynamic Therapy
Source: Pharmaceutics. 2022 Aug 7;14(8):1645. doi: 10.3390/pharmaceutics14081645 (PMC9416189; doi:10.3390/pharmaceutics14081645)
Supplement: Supplementary file 1 [file pharmaceutics-14-01645-s001.zip › pharmaceutics-1805251-supplementary.pdf]

Supporting Information

Gold Nanoclusters-Based NIR-II Photosensitizers with Catalase-like Activity for Boosted Photodynamic Therapy

Qing Dan [1,2], Zhen Yuan [3], Si Zheng [1,2], Huanrong Ma [1], Wanxian Luo [1], Li Zhang [1], Ning Su [1], Dehong Hu [2], Zonghai Sheng [2,*] and Yingjia Li [1,*]

[1] Department of Medicine Ultrasonics, Nanfang Hospital, Southern Medical University, Guangzhou 510515, China
[2] Shenzhen Institute of Advanced Technology, Chinese Academy of Sciences, Shenzhen 518055, China
[3] Faculty of Health Sciences, University of Macau, Taipa, Macau SAR, China
* Correspondence: zh.sheng@siat.ac.cn (Z.S.); lyjia@smu.edu.cn (Y.L.)

Content

Experimental section 3

Supplementary tables 9

Table S1. The preparation optimization of BSA@Au 9

Table S2. Comparison of kinetic parameters among nanozymes 10

Table S3. Blood sample examination of mice.. 11

Supplementary figures 12

Figure S1. Optimization of BSA@Au 12

Figure S2. Fluorescent QY of BSA@Au 13

Figure S3. Penetration depth of NIR-I and NIR-II FL imaging.......................... 14

Figure S4. Photostability of BSA@Au 15

Figure S5. Catalase-like activity of BSA@Au 16

Figure S6. Biosafety of BSA@Au... 17

Figure S7. *Ex vivo* FL images 18

Figure S8. Temperature of mice during cancer PDT 19

Figure S9. Tumor inhibition of mice received different treatments.................... 20

Figure S10. Body weights of mice during cancer PDT 21

Figure S11. *In vivo* NIR-II FL imaging for MRSA-infected mice...................... 21

Figure S12. Body weights of mice during MRSA PDT...................................... 22

Figure S13. Temperature of mice during MRSA PDT.. 22

References 23

Experimental section

Characterization of BSA@Au. UV-Vis absorption spectra and fluorescence (FL) emission spectra were determined by a UV-Vis spectrometer (Lambda 25 UV/VIS Spectrometer, PerkinElmer, America) and a FL spectrometer (FLS920, Edinburgh Instruments, UK), respectively. High resolution transmission electron microscopy (Jem-2100F, Jeol, Japan) was used to obtain the structural and morphological details of the BSA@Au. Zetasizer (NanoZS, Marlvern, UK) was used to determine the hydrophilic size of BSA@Au.

Photostability of BSA@Au. BSA@Au and indocyanine green (ICG, 10 μg/mL) were respectively exposed to laser irradiation (808 nm, 0.3 W/cm^2). Then, the FL intensity of BSA@Au and ICG solutions was measured. The average FL intensity of the region of interest was plotted as a function of time.

Penetration depth. Penetration depth measurement of BSA@Au was studied in a simulated deep tissue setting. BSA@Au solution in eppendorf tube was covered by chicken-breast tissues with various thickness. The NIR-I FL images (λ_{ex} = 675 nm, λ_{em} = 800 nm) and NIR-II FL images (λ_{em} = 808 nm, 1000 nm long-pass filter, 30 ms) were taken.

Catalase-like activity of BSA@Au. Referring to our previous study [1],we designed a semi-sealed chamber equipped with a dissolved oxygen probe (DO200, Clean, China) to measure the amount of O_2 production, which could verify the catalase-like activity

of BSA@Au to decompose H_2O_2 to O_2. In brief, N_2 was introduced into BSA@Au solution (1 mL) to remove the pre-dissolved O_2. Then, H_2O_2 solution (1 mL) were softly added into the semi-sealed reaction chamber. Every 30 s, the dissolved O_2 was measured. According to the Michaelis-Menten equation [2], the apparent kinetic parameters were obtained to evaluate the catalytic performance of BSA@Au:

$$V = (V_{max} \times [S])/(K_m + [S])$$

The V, V_{max}, [S], and K_m denoted the initial reaction velocity, the maximal reaction velocity, the substrate (H_2O_2) concentration, and the Michaelis-Menten constant, respectively. We further calculated the V_{max} and K_m values from the Lineweaver-Burk plot method:

$$V^{-1} = K_{max}/(V_{max} \times [S]) + V_{max}^{-1}$$

Next, Ultrasound imaging system (VEVO 2100, FUJIFILM Visual Sonics, US) was used to detect the signal of bubbles generated by the reaction between BSA@Au and H_2O_2. The imaging conditions were set conformably (frequency: 18 MHz; 2D gain: 18 dB; and CEUS gain: 30 dB).

QY of BSA@Au. QY of BSA@Au was measured against the dye IR-26 (QY ≈ 0.5%) [3]. Generally, IR-26 was freshly dissolved with 1,2-dichlorethane (1,2-DCE), and the absorbance values of 0.023, 0.043, 0.067, 0.084, and 0.109 at 808 nm were respectively obtained. Similarly, BSA@Au solution was diluted to various concentrations with absorbance of 0.024, 0.044, 0.061, 0.081, and 0.099 at 808 nm. For IR-26 and BSA@Au, the integrated total emission intensity in the 900 ~ 1500 nm region was

collected under an excitation wavelength of 808 nm. Next, the linear functions were obtained by plotting the integrated FL intensity against the absorption intensity at 808 nm. Finally, the QY of BSA@Au was calculated according to the following equation [4]:

$$QY_{BSA@Au} = QY_{IR\text{-}26} \times (S_{BSA@Au}/S_{IR\text{-}26}) \times (\eta_{BSA@Au}/\eta_{IR\text{-}26})^2$$

The S represented the slope of the integrated FL against absorbance at 808 nm, and the η denoted the refractive index of water for BSA@Au (η ≈ 1.3) and 1, 2-DCE for IR-26 (η ≈ 1.4), respectively.

In vitro **$^1O_2$ generation.** $^1O_2$ generation ability of BSA@Au was determined with DPBF as $^1O_2$ indicator and ICG as reference. Briefly, ICG (10 μg/mL) or BSA@Au (0.1 mM) were mixed with DPBF, followed by laser irradiation (808 nm, 0.3 W/cm^2). The absorbance at 414 nm was recorded every 2 minutes. The $^1O_2$ generation efficiency of BSA@Au ($\Phi_{\Delta BSA@Au}$) was calculated according to the equation [4]:

$$\Phi_{\Delta BSA@Au} = (S_{BSA@Au}/S_{ICG}) \times \Phi_{\Delta ICG}$$

The S indicated the slope of absorbance at 414 nm against time, and the $\Phi_{\Delta ICG}$ was reported as 0.2%.

To evaluate the $^1O_2$ generation of BSA@Au enhanced by O_2, H_2O_2 was introduced to produce O_2 catalyzed by BSA@Au. When BSA@Au solutions were mixed with H_2O or H_2O_2, the DPBF was immediately added to the mixtures to measure $^1O_2$ under laser illumination (808 nm, 0.3 W/cm^2).

Cell line, MRSA strain, and animal models. The 4T1 cell lines were cultured in DMEM medium at 37 °C in an atmosphere containing 5% CO_2. Methicillin-resistant staphylococcus aureus (MRSA, ATCC 43300 Strains) were cultured with Luria-Bertani medium at 37 °C.

The animal experiments were strictly conducted in accordance with the ethical guidelines for animal research of the Institutional Animal Care and Use Committee of Shenzhen Institute of Advanced Technology. Balb/C nude mice and Balb/C mice (female, 6 ~ 8 weeks) were used to establish breast tumor-bearing mouse models. Subcutaneous tumors were generated by implanting 4T1 cells (1×10^6 cells, 100 μL) suspended in serum-free DMEM medium into the right hind legs of the mice. Tumor volume (V) was calculated according to the formula:

$$V = (\text{Length} \times \text{Width}^2)/2$$

When the V reached about 50 ~ 100 mm^3, the FL imaging, ultrasound imaging, and photodynamic therapy were carried out, respectively.

To establish the MRSA-infected mouse models, dorsal skin of the Balb/C mice was firstly removed by ophthalmic scissors to form round wounds (wound diameter: 8 mm). Then, the established wounds were subcutaneously injected 50 μL of MRSA suspension ($OD_{600\ nm} \approx 1$). After 24 h, the infected wounds were generated for further investigation.

Cytotoxicity of BSA@Au. To determine the cytotoxicity of BSA@Au, the 4T1 cells (1×10^4 cells per well) were seeded in 96-well plates. After 12 h cultivation, the medium was replaced with fresh medium containing different concentrations of BSA@Au for 24 h incubation. Then, cell viabilities were determined by CCK-8 assay kit.

Hemolysis assay. The whole blood samples of Balb/C mice were collected and centrifuged with a low speed (2,000 rpm, 3 min). Then, the red blood cells

(RBCs) were washed with PBS for three times. Next, RBCs suspensions were further incubated with BSA@Au of various concentrations (20, 40, 75, 150, 300, 625, and 1250 μg/mL) at 37 °C for 2 h. Then, the suspensions were centrifugated (5, 000 rpm, 2 min). The supernatants were collected to determine the absorbance of released hemoglobin at 540 nm. The hemolytic rate (HR) was calculated referring to the equation:

$$HR\ (\%) = (A_{BSA@Au} - A_n)/(A_p - A_n) \times 100\%$$

The $A_{BSA@Au}$, A_p, and A_n denoted the absorbance of BSA@Au group, the positive control group (RBCs in pure water) at 540 nm, and the negative control group (RBCs in PBS), respectively.

***In vitro* anti-tumor PDT.** The 4T1 cells (5, 000 cells per well) were seeded in a 96-well plate. After 12 h incubation with BSA@Au (100 μg/mL), the cells were washed with PBS and irradiated with 808 nm laser (0.3 W/cm^2, 30 min) in an ice bath to avoid the photothermal effect. Then, the cells were incubated for 4 h, and relative cell viability was analysed by the CCK-8 method. Furthermore, the *in vitro* PDT effect was verified via live (calcein-AM)/dead (PI) double-staining. The 4T1 cells (5 × 10^4 cells per well) were seeded in a 6-well plate. After 12 h incubation with BSA@Au (100 μg/mL), the cells were washed with PBS and irradiated with 808 nm laser (0.3 W/cm^2, 30 min) in an ice bath. After incubation for 4 h, the cells were then stained with calcein-AM (2 μM) and PI (4.5 μM) and observed under a biological inverted microscope (Olympus IX71, Olympus, Japan).

***In vivo* ultrasound imaging.** As for ultrasound imaging, Balb/C mice received intratumoral injection of PBS or BSA@Au (2.5 mM, 50 μL), respectively.

Hypoxia modulation. To evaluate the *in vivo* tumor hypoxia modulation ability, Pimonidazole HCl was used as the hypoxia probe to observe the hypoxic tumor areas by immunofluorescence staining. The 4T1 tumor-bearing mice were intravenously injected with BSA@Au (0.46 mM,100 μL) and PBS (100 μL), respectively. After 10 h, the mice received intraperitoneal injection of Pimonidazole HCl with a dosage of 60 mg/Kg. Approximately 90 min later, tumors were collected for frozen sections. Then, following manufacturer's instructions, the tumor frozen sections incubated with FITC-conjugated IgG_1 mouse monoclonal antibody (1:50 dilution) and stained with DAPI. Then, the stained tumor sections were observed under an inverted microscope (Olympus IX71, Olympus). Quantification of the hypoxia area was realized by ImageJ software and calculated according to this equation:

$$\text{Hypoxia positive area} = (\text{Hypoxic area} / \text{Total area}) \times 100\%$$

***In vivo* toxicity.** To assess the *in vivo* biosafety of BSA@Au, healthy Balb/C mice (n = 3) received intravenous injection of BSA@Au (5 mM, 100 μL). After 14 days, whole blood samples were collected and analyzed by blood routine examination.

Supplementary Tables

Table S1. The preparation optimization of BSA@Au by mixing different concentrations and volumes of BSA, $HAuCl_4$, NaOH, and $NaBH_4$. According to the FL intensity upon NIR-II FL imaging, sample 3 was chosen for further investigation.

**Sample**	**BSA (50 mg/mL)**	**$HAuCl_4$ (10 mM)**	**NaOH (1 M)**	**$NaBH_4$ (0.1 M)**	**pH**	**NIR-II emission intensity**
1	2.5 mL	2.5 mL	0.25 mL	0.25 mL	~ 11	+
2	2.5 mL	0.75 mL	0.25 mL	0.25 mL	~ 11	++
**3**	**2.5 mL**	**1.25 mL**	**0.25 mL**	**0.1 mL**	**~ 11**	**++++**
4	0.75 mL	2.5 mL	0.25 mL	0.1 mL	~ 12	++
5	2.5 mL	0.75 mL	0.5 mL	0.05 mL	~ 12.4	+
6	2.5 mL	0.75 mL	1 mL	0.05 mL	~ 12.8	+
7	2.5 mL	1.25 mL	0.25 mL	/	~ 11	/

Table S2. Comparison of kinetic parameters among nanozymes. V_{max} represented the reaction activity, and K_m indicated the affinity of the enzyme towards the substrate, respectively. The higher V_{max} value, the greater catalase-like activity. The lower K_m value, the stronger the affinity.

**Nanozymes**	**K_m/mM**	**V_{max}/ mM s^{-1}**	**References**
Co_3O_4 (nanoplates)	24.7	2.38×10^{-3}	
Co_3O_4 (nanorods)	4.82	1.89×10^{-3}	[5]
Co_3O_4 (nanocubes)	63.9	1.23×10^{-3}	
Pt-Ft	420.6	0.84	[6]
Ac-G9/Pt	126.1	8.249×10^{-2}	[7]
RuO_2	400	9.68×10^{2}	[8]
PVP-IrNPs	297	8.249×10^{-2}	[9]
AuNCs-NH_2 (pH 7.4)	10.6	3.91×10^{-4}	[10]
Au NCs-ICG	2.02	4.55×10^{-3}	[1]
**BSA@Au**	**8.32**	**9.59×10^{-4}**	**This work**

Table S3. Blood sample examination of mice (n = 3) at 14 days after i.v. injection of BSA@Au (5 mM, 100 μL).

Item	Value	References
RBC (10^{12}/L)	9.25 ± 0.06	6.36 ~ 9.42
WBC (10^{9}/L)	4.00 ± 0.26	0.60 ~ 6.80
Lymph (10^{9}/L)	3.10 ± 0.20	0.70 ~ 5.70
Mon (10^{9}/L)	0.10	0.00 ~ 0.30
Gran (10^{9}/L)	0.73 ± 0.06	0.10 ~ 1.80
Lymph (%)	76.03 ± 1.62	55.80 ~ 90.60
Mon (%)	3.93 ± 0.57	1.80 ~ 6.00
Gran (%)	20.03 ± 2.10	8.60 ~ 38.9
HGB (g/L)	142 ± 3	110 ~ 143
HCT (%)	42.53 ± 1.36	34.6 ~ 44.6
MCV (fL)	55.20 ± 0.92	48.2 ~ 58.3
MCH (pg)	14.63 ± 0.23	15.80 ~ 19.00
MCHC (g/L)	263.00 ± 4.58	302 ~ 353
RDW (%)	14.97 ± 0.25	13 ~ 17

Supplementary figures

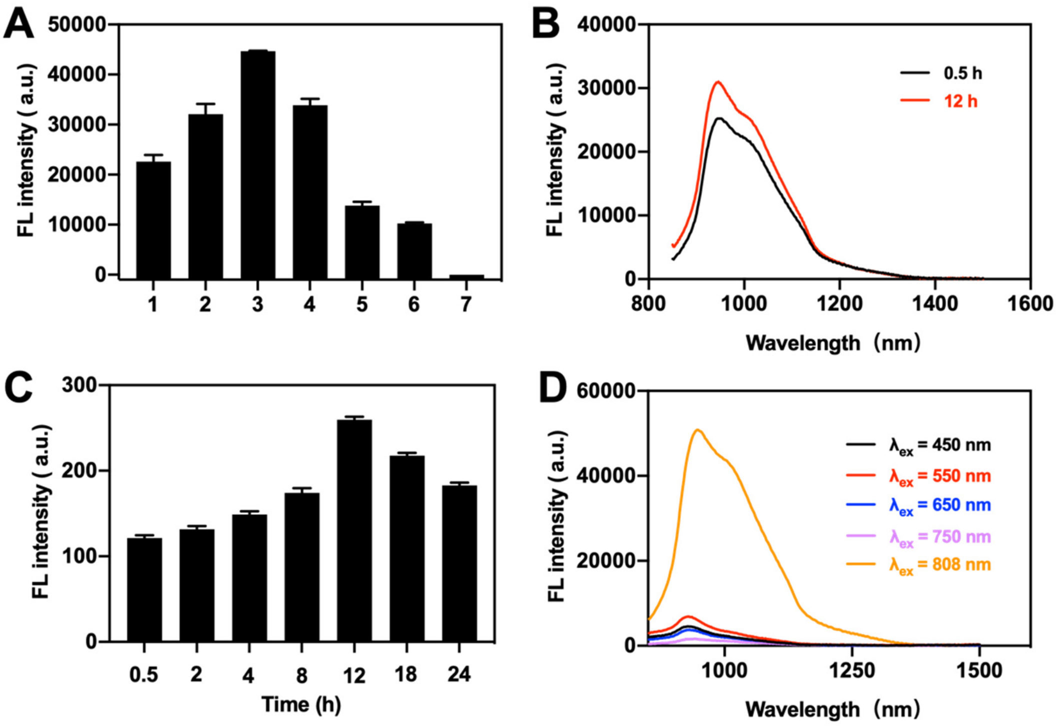

Figure S1. Optimization of BSA@Au. (A) The quantification of FL intensity of BSA@Au samples 1-7 prepared under different conditions (samples 1-7 preparation details seen Table S1). (B) FL spectra of BSA@Au under different incubation time (0.5 h and 12 h). (C) Quantification of NIR-II FL intensity of BSA@Au under different incubation time (0.5, 2, 4, 8, 12, 18, and 24 h). (D) Excitation wavelength-dependent FL emission of BSA@Au.

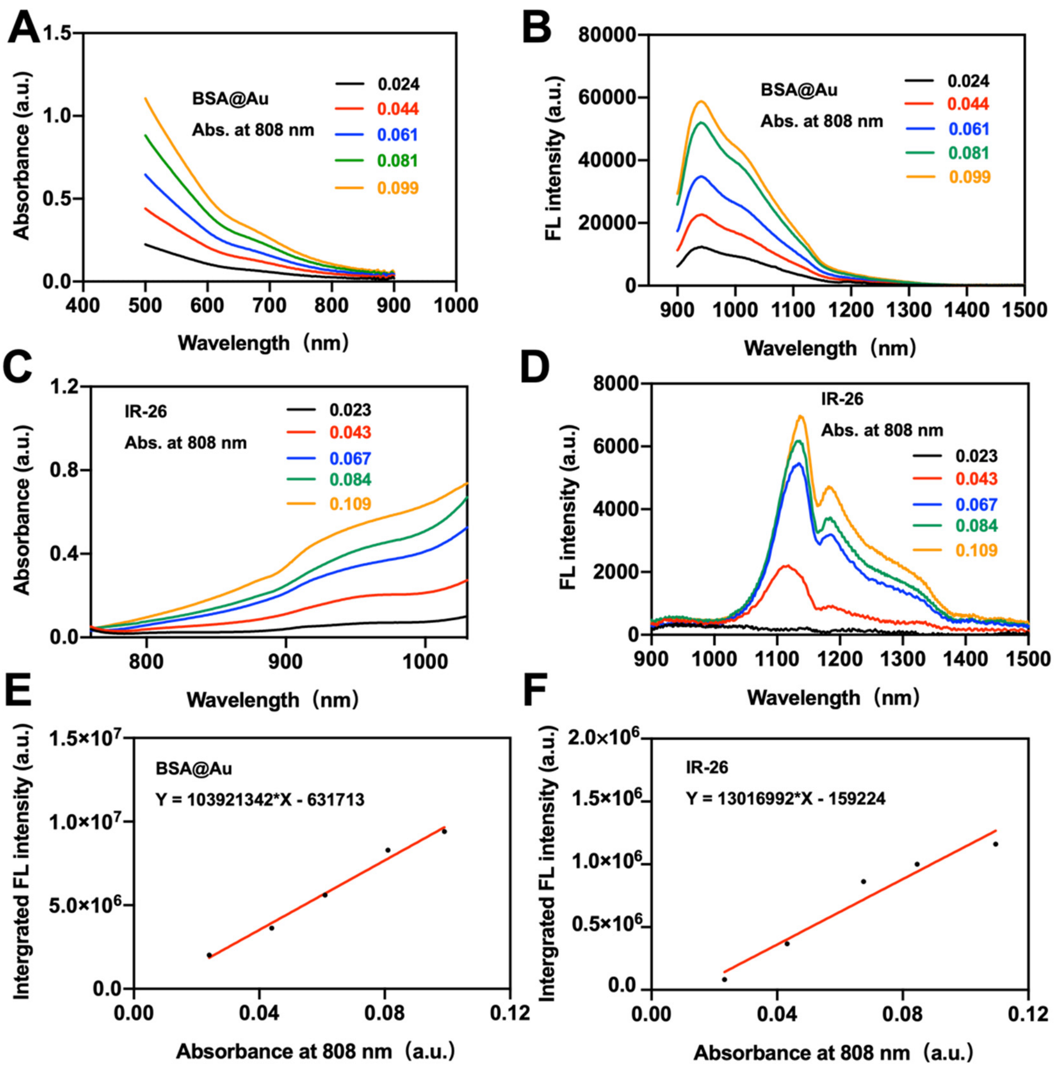

Figure S2. Fluorescent QY of BSA@Au. (A) Absorption of BSA@Au with different concentrations. (B) NIR FL emission spectra of BSA@Au with different concentrations under 808 nm excitation. (C) Absorption spectra of IR-26 in DCE with different concentrations. (D) NIR FL emission spectra of IR-26 in DCE with different concentrations under 808 nm excitation. (E) The absorbance values of BSA@Au in water with different concentrations were plotted versus area under curve (AUC) and fitted into a linear function. (F) The absorbance values of IR-26 in DCE with different concentrations were plotted versus AUC and fitted into a linear function.

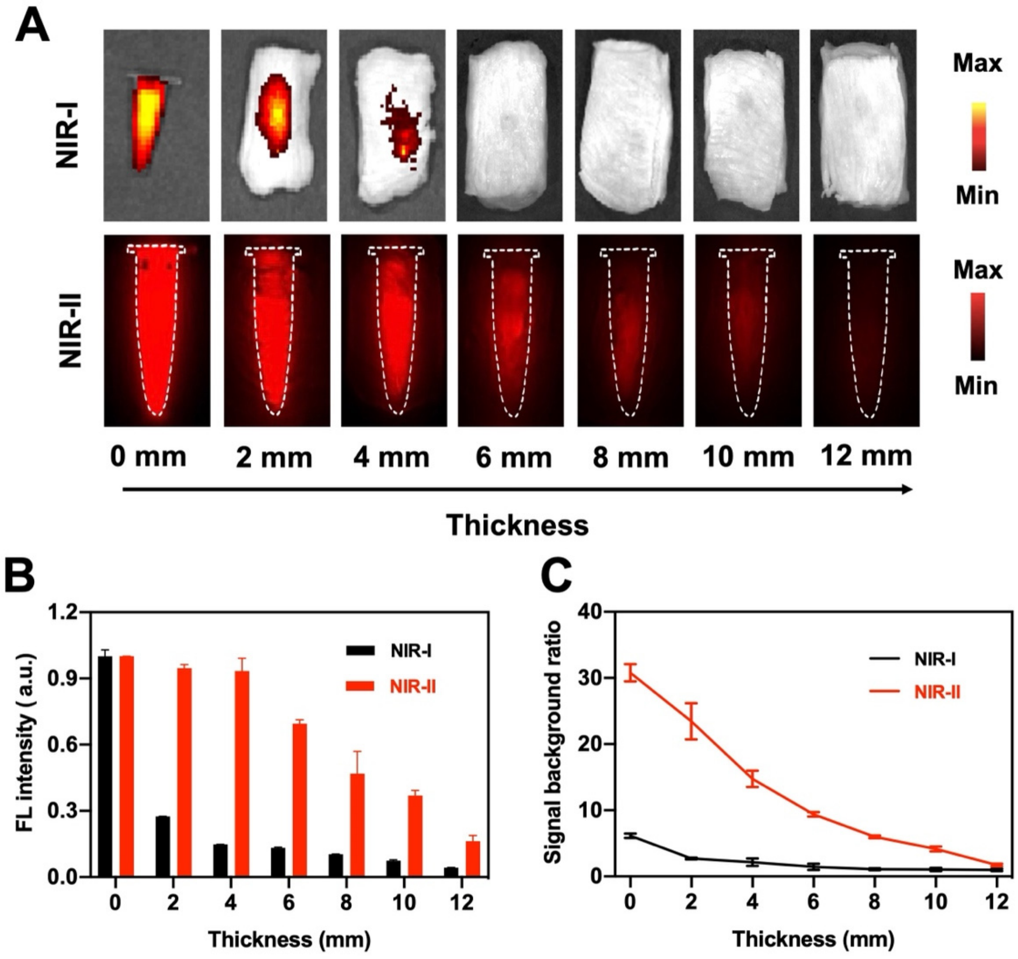

Figure S3. Penetration depth of NIR-I and NIR-II FL imaging. (A) Comparison of tissue penetration depth of BSA@Au measured by NIR-I and NIR-II FL imaging system. (B) Quantification of FL signal intensity against chicken tissue thickness shown in (A). (C) The signal to background ratio of BSA@Au covered by chicken tissues of various thickness in NIR-I and NIR-II FL imaging.

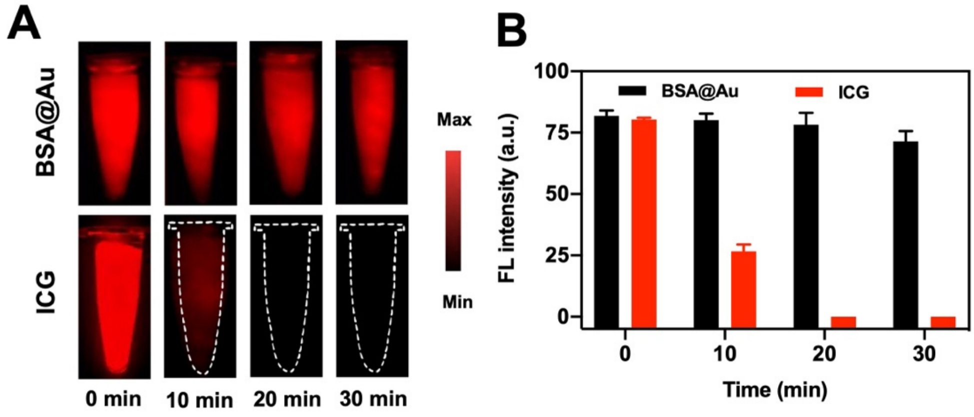

Figure S4. Photostability of BSA@Au. (A) Photostability of BSA@Au and ICG after laser irradiation (808 nm, 0.3 W/cm^2). (B) Quantification of NIR-II FL signal intensity shown in (A).

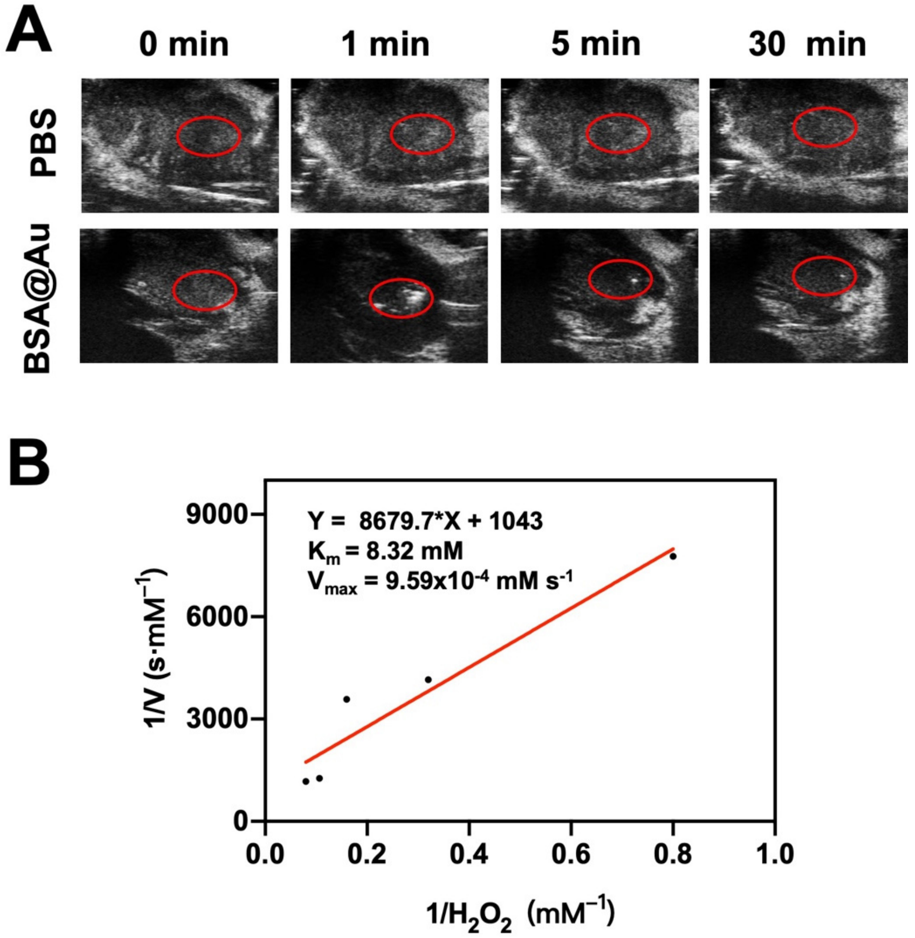

Figure S5. Catalase-like activity of BSA@Au. (A) Ultrasound imaging of bubbles generation after intratumoral injection of PBS or BSA@Au. (B) Double-reciprocal plots of activities of BSA@Au which derived from the Michaelis-Menten equation.

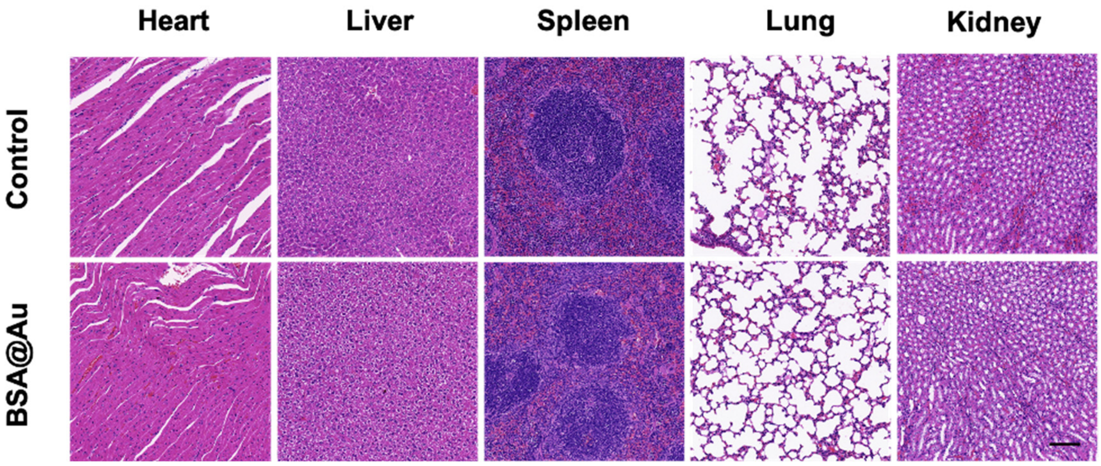

Figure S6. Biosafety of BSA@Au. H&E staining assay of main organs (including heart, liver, spleen, lung, and kidneys) from mice intravenously injected with BSA@Au (5 mM, 100 μL) and PBS for 14 days.

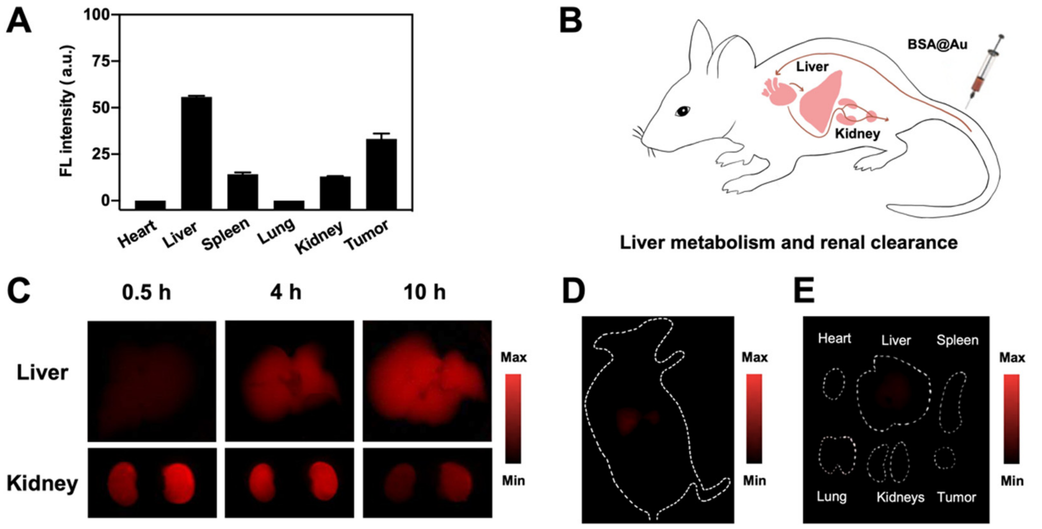

Figure S7. *Ex vivo* NIR-II FL imaging. (A) The quantification of *ex vivo* NIR-II FL images of main organs (heart, liver, spleen, lung, and kidneys) and tumor harvested from 4T1 tumor-bearing mice at 10 h post injection of BSA@Au (0.46 mM, 100 μL). (B) The schematic illustration of metabolism and renal clearance of BSA@Au. (C) The real-time NIR-II FL images of liver and kidneys post injection of BSA@Au (0.46 mM, 100 μL). (D) *In vivo* NIR-II FL images of 4T1 tumor-bearing mice at 48 h post injection of BSA@Au. (E) *Ex vivo* NIR-II FL images of main organs (heart, liver, spleen, lung, and kidneys) and tumor harvested from 4T1 tumor-bearing mice at 48 h post injection of BSA@Au.

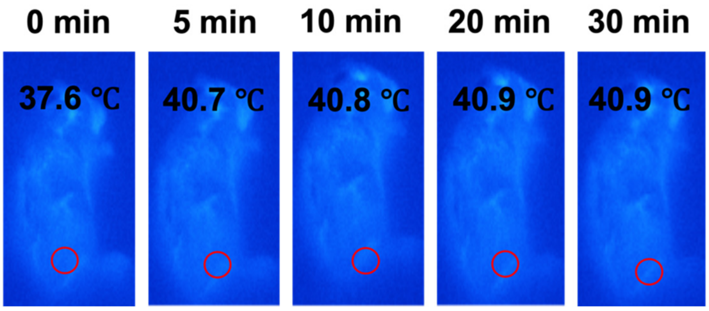

Figure S8. Temperature changes of 4T1 tumor-bearing mice recorded during PDT.

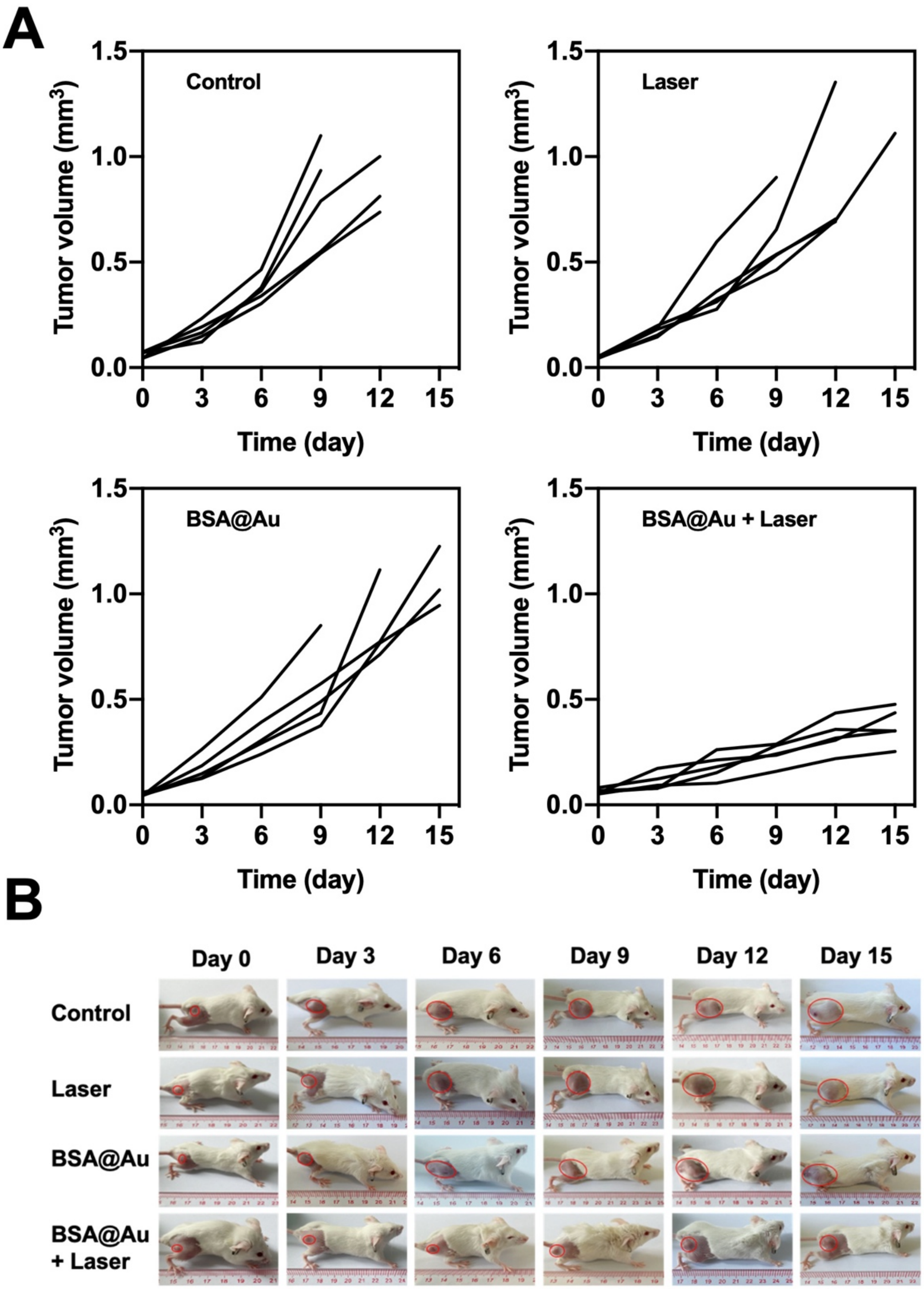

Figure S9. Tumor inhibition of mice received different treatments. (A) Tumor inhibition of mice received different treatments. (B) Representative photos of 4T1 tumor-bearing mice after *in vivo* PDT treatments over time.

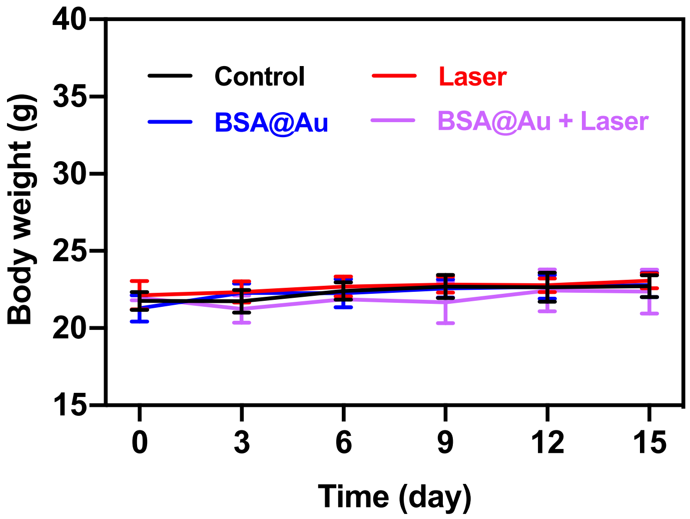

Figure S10. The body weight changes of 4T1 tumor-bearing mice over time during the PDT.

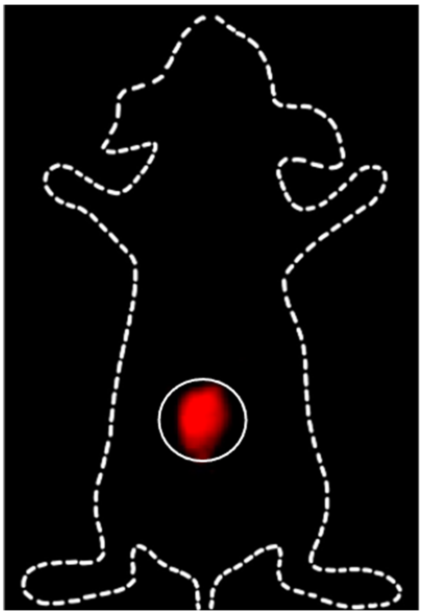

Figure S11. *In vivo* NIR-II FL imaging for MRSA-infected mice.

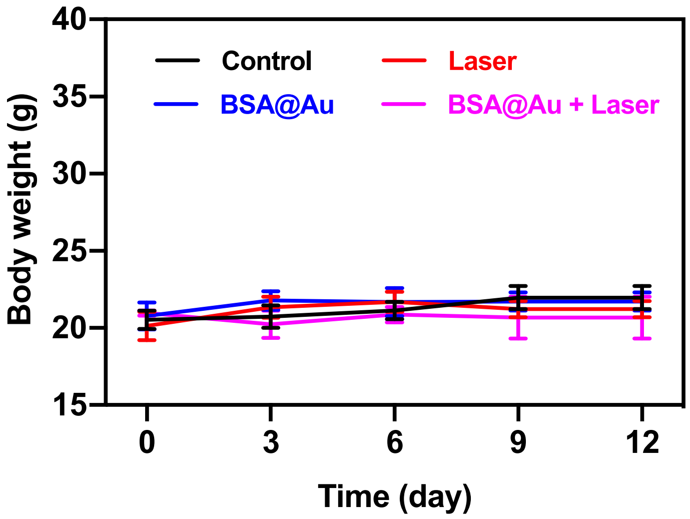

Figure S12. The body weight changes of MRSA-infected mice over time during the PDT.

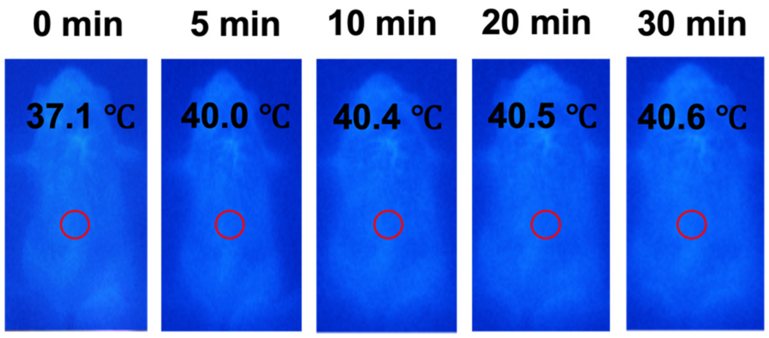

Figure S13. Temperature changes of MRSA-infected mice recorded during PDT.

References

1. Dan, Q.; Hu, D.; Ge, Y.; Zhang, S.; Li, S.; Gao, D.; Luo, W.; Ma, T.; Liu, X.; Zheng, H.; et al. Ultrasmall Theranostic Nanozymes to Modulate Tumor Hypoxia for Augmenting Photodynamic Therapy and Radiotherapy. *Biomater. Sci.* **2020**, *8*, 973–987, doi:10.1039/C9BM01742A.
2. Johnson, K.A.; Goody, R.S. The Original Michaelis Constant: Translation of the 1913 Michaelis–Menten Paper. *Biochemistry* **2011**, *50*, 8264–8269, doi:10.1021/bi201284u.
3. Murphy, J.E.; Beard, M.C.; Norman, A.G.; Ahrenkiel, S.P.; Johnson, J.C.; Yu, P.; Mićić, O.I.; Ellingson, R.J.; Nozik, A.J. PbTe Colloidal Nanocrystals: Synthesis, Characterization, and Multiple Exciton Generation. *J. Am. Chem. Soc.* **2006**, *128*, 3241–3247, doi:10.1021/ja0574973.
4. Wang, Q.; Xu, J.; Geng, R.; Cai, J.; Li, J.; Xie, C.; Tang, W.; Shen, Q.; Huang, W.; Fan, Q. High Performance One-for-All Phototheranostics: NIR-II Fluorescence Imaging Guided Mitochondria-Targeting Phototherapy with a Single-Dose Injection and 808 nm Laser Irradiation. *Biomaterials* **2020**, *231*, 119671, doi:10.1016/j.biomaterials.2019.119671.
5. Mu, J.; Zhang, L.; Zhao, M.; Wang, Y. Catalase Mimic Property of Co_3O_4 Nanomaterials with Different Morphology and Its Application as a Calcium Sensor. *ACS Appl. Mater. Interfaces* **2014**, *6*, 7090–7098, doi:10.1021/am406033q.
6. Fan, J.; Yin, J.-J.; Ning, B.; Wu, X.; Hu, Y.; Ferrari, M.; Anderson, G.J.; Wei, J.; Zhao, Y.; Nie, G. Direct Evidence for Catalase and Peroxidase Activities of Ferritin-Platinum Nanoparticles. *Biomaterials* **2011**, *32*, 1611–1618, doi:10.1016/j.biomaterials.2010.11.004.
7. Wang, X.; Zhang, Y.; Li, T.; Tian, W.; Zhang, Q.; Cheng, Y. Generation 9 Polyamidoamine Dendrimer Encapsulated Platinum Nanoparticle Mimics Catalase Size, Shape, and Catalytic Activity. *Langmuir* **2013**, *29*, 5262–5270, doi:10.1021/la3046077.
8. Deng, H.; Shen, W.; Peng, Y.; Chen, X.; Yi, G.; Gao, Z. Nanoparticulate Peroxidase/Catalase Mimetic and Its Application. *Chem. - Eur. J.* **2012**, *18*, 8906–8911, doi:10.1002/chem.201200643.
9. Su, H.; Liu, D.-D.; Zhao, M.; Hu, W.-L.; Xue, S.-S.; Cao, Q.; Le, X.-Y.; Ji, L.-N.; Mao, Z.-W. Dual-Enzyme Characteristics of Polyvinylpyrrolidone-Capped Iridium Nanoparticles and Their Cellular Protective Effect against H_2O_2 -Induced Oxidative Damage. *ACS Appl. Mater. Interfaces* **2015**, *7*, 8233–8242, doi:10.1021/acsami.5b01271.
10. Liu, C.-P.; Wu, T.-H.; Liu, C.-Y.; Chen, K.-C.; Chen, Y.-X.; Chen, G.-S.; Lin, S.-Y. Self-Supplying O_2 through the Catalase-Like Activity of Gold Nanoclusters for Photodynamic Therapy against Hypoxic Cancer Cells. *Small* **2017**, *13*, 1700278, doi:10.1002/smll.201700278.
